# The effect of fenestration of the lamina terminalis on the incidence of shunt-dependent hydrocephalus after aneurysmal subarachnoid hemorrhage (FISH): Study protocol for a randomized controlled trial

**DOI:** 10.1097/MD.0000000000005727

**Published:** 2016-12-30

**Authors:** Chuanyuan Tao, Chaofeng Fan, Xin Hu, Junpeng Ma, Lu Ma, Hao Li, Yi Liu, Hong Sun, Min He, Chao You

**Affiliations:** Stroke Clinical Research Unit, Department of Neurosurgery, West China Hospital, Sichuan University, Chengdu, P. R. China.

**Keywords:** aneurysmal subarachnoid hemorrhage, fenestration of laminal terminalis, outcomes, prognosis, shunt-dependent hydrocephalus

## Abstract

**Background:**

Shunt-dependent hydrocephalus (SDH) is a well-known sequela following aneurysmal hemorrhage, adversely affecting the outcome after securing ruptured aneurysm. Fenestration of lamina terminalis (FLT) creates an anterior ventriculostomy, facilitates cerebrospinal fluid circulation and clot clearance in the basal cistern. However, controversy exists over whether microsurgical FLT during aneurysm repair can decrease the incidence of SDH.

**Aims:**

The study is designed to determine the efficacy of lamina terminalis fenestration on the reduction of SDH after aneurysm clipping.

**Methods/Design:**

A total of 288 patients who meet the inclusion criteria will be randomized into single aneurysm clipping or aneurysm clipping plus FLT in the Department of Neurosurgery, West China Hospital. Follow-up was performed 1, 3, 6, and 12 months after aneurysm clipping. The primary outcome is the incidence of SDH and the secondary outcomes include cerebral vasospasm, functional outcome evaluated by the modified Rankin Scale and Extended Glasgow Outcome Scale, and mortality.

**Discussion:**

The FISH trial is a large randomized, parallel controlled clinical trial to define the therapeutic value of FLT, the results of which will help to guide the surgical procedure and resolve the long-puzzled debate in the neurosurgical community.

**Conclusions:**

This protocol will determine the efficacy of FLT in the setting of aneurysmal subarachnoid hemorrhage.

**Trial registration identifier:**

http://www.chictr.org.cn/edit.aspx?pid=15691&htm=4

**Chinese Clinical Trial Registry:**

ChiCTR-INR-16009249.

## 1 Background

Subarachnoid hemorrhage (SAH) is a major subset of cerebral hemorrhagic stroke with an annual incidence of 2 to 32 cases per 100,000 population.^[1]^ The vast majority of SAH (>85%) are caused by ruptured aneurysm.^[2,3]^ Aneurysmal SAH (aSAH) is a fetal condition with which 15% patients die on the spot,^[4]^ 40% within a month,^[5]^ only 55% patients are independent among survivors,^[4]^ and survivors of aSAH after 20 years showed 17% excess mortality compared with the general population.^[6]^ Surgical clipping to isolate the blood circulation of aneurysm cavity with parental artery is regarded as the treatment of choice although endovascular coiling with or without stent is getting popular.

Many a factors are associated with a poor outcome after aSAH, one of which is shunt-dependent hydrocephalus (SDH). In general, aSAH-induced hydrocephalus can be categorized as either acute or chronic considering the time course to presentation.^[7–9]^ Acute hydrocephalus occurs within hours mainly due to blockage of CSF flow^[8]^ while time of chronic hydrocephalus can vary from weeks to months and is not necessarily preceded by acute hydrocephalus.^[9]^ For patients with acute hydrocephalus, external ventricular drainage is performed to temporarily bypass cerebrospinal fluid. Most of these patients can tolerate clamping of the drainage at later stage during hospitalization while approximately 30% patients need permanent shunt.^[10,11]^ Meanwhile, the incidence of chronic hydrocephalus requiring shunt surgery was reported more than 20%.^[12–14]^ Therefore, SDH is a common complication following aSAH. According to the recent studies, the incidence of SDH after aSAH was around 20%^[13,15,16]^ ranging from 17.2% to 31.2%.^[17–19]^ Patients with SDH experienced higher mortality, worse short- and long-term prognosis with longer hospital stay.^[20,21]^ Moreover, SDH is an independent risk factor predicting long-term unfavorable functional outcome of aSAH.^[20]^

Traditionally, ventriculoperitoneal shunt is the treatment of choice after SDH. However, CSF shunts have high malfunction rates with reported 43% and 85% of shunts failure at 1 and 10 years after the first placement, respectively.^[22]^ Moreover, the procedure of shunt placement or revision has the risk of intracerebral hemorrhage,^[23]^ CSF leakage, infection, and bowel perforation or pneumothorax, with mortality up to 7% to 9% in selective populations.^[22,24,25]^ Specifically, although SDH after aSAH was considered less frequently to suffer shunt failure compared with other forms of hydrocephalus, 30% patients required a subsequent revision which contributed to multiple revisions.^[15]^

Fenestration of lamina terminalis (FLT) is a useful procedure during ruptured aneurysm surgical clipping to allow brain relaxation under increased intracranial pressure by offering free CSF flow from the third ventricle to the basal cistern. In addition, FLT has been performed to prevent the occurrence of SDH through facilitating CSF circulation, clearance of blood in basal cisterns, and improving CSF dynamics.^[8]^ Tomasello et al^[26]^ first assessed the effect of FLT in preventing SDH in 1999. Of the 52 patients included, only 2 (4.2%) patients developed SDH with a follow-up of 12 to 60 months. Since then, more than 400 patients were treated combining FLT, clot evacuation from the basal cisterns, and opening of the Liliequist membrane with the incidence of SDH less than 4%. Komotar et al^[27]^ in 2002 further confirmed the usefulness of FLT in a large population (582 cases) as they showed a decreased incidence of SDH in more than 80% patients with aSAH. Moreover, it was demonstrated that FLT was not also associated with reduced SDH, but also with decreased vasospasm and better outcome.^[28]^ Hence, FLT was recommended as a routine practice in patients with severe aSAH.^[27]^

However, the therapeutic role of FLT is questioned by recent studies. Komotar et al^[29]^ failed to observe decreased SDH as fenestrated patients had a shunt rate of 25% compared with 20% in unfenestrated patients. The negative results were supported by Chohan et al^[30]^ who intraventricularly injected a contrast agent to evaluate the functional patency of the fenestrated lamina terminalis with CT imaging, finding that contrast agent followed the normal ventricular pathway into fourth ventricle rather than in the basal cisterns. To define the controversial role of FLT, a systematic review including 11 studies involving 1973 patients was performed where no significant association between FLT and a reduced incidence of SDH was revealed with an overall incidence of 10%, 14% in the fenestrated and nonfenestrated cohort, respectively.^[13]^ However, the baseline data between the 2 cohorts were inconsistent, especially with more patients in poor clinical condition in the former. Actually, high grade SAH is a risk factor of the occurrence of SDH,^[17,18]^ so this difference reinforced the efficacy of FLT. Therefore, a well-designed randomized controlled trial is widely called on to definitively determine the efficacy of this technique.

To the best of our knowledge, only a single randomized controlled study was performed to address such debate.^[7]^ This clinical trial included 50 patients with ruptured anterior communicating aneurysm, suggested no significant difference between fenestrated and nonfenestrated group (24% vs 16%). However, more than 90% patients with low grade SAH and about 60% with Fisher grade I/II were included which underpowered to detect the difference as patients with low grade of H-H Grade or Fisher Grade are quite less likely to develop SDH. In addition, this study was obviously limited by the quite small sample size.

We designed this study to provide more robust evidence of the efficacy of FLT during surgical aneurysm clipping on the prevention of SDH with a large sample size and strict inclusion and exclusion criteria.

## 2 Methods/design

### 2.1 Study design

**Figure 1:**
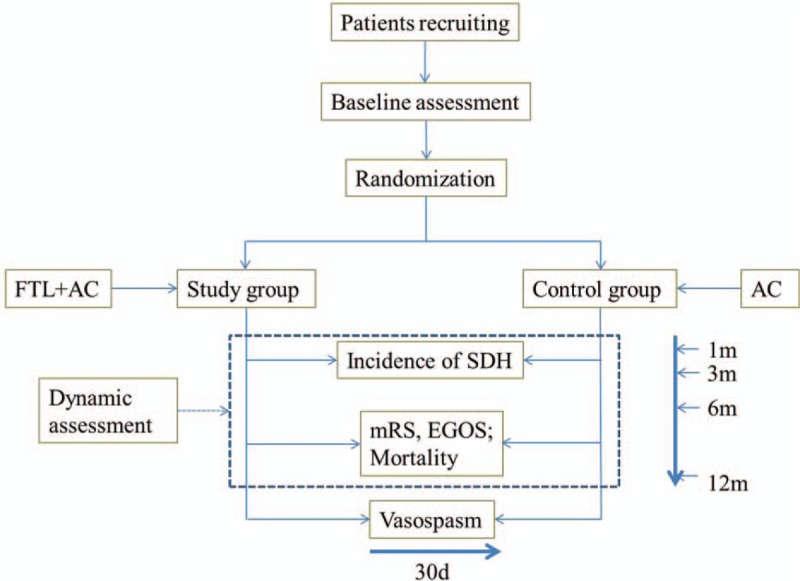
Flow chart of the clinical trial. AC = aneurysm clipping, EGOS = extended Glasgow outcome scale, FLT = fenestration of lamina terminalis, mRS = modified Rankin Scale, SDH = shunt-dependent hydrocephalus.

The FISH trial is a prospective, single-center, parallel, randomized, assessor-blinded trial. The overall flowchart is demonstrated in Figure 1. The main objective is to compare the incidence of SDH in the patients undergoing aneurysm clipping plus FLT or not. The secondary aim is to determine the effects of FLT on vasospasm within 1 month and functional outcome within 1 year in patients with anterior circulation aneurysm.

### 2.2 Ethics approval and consent to participate

**Figure 2:**
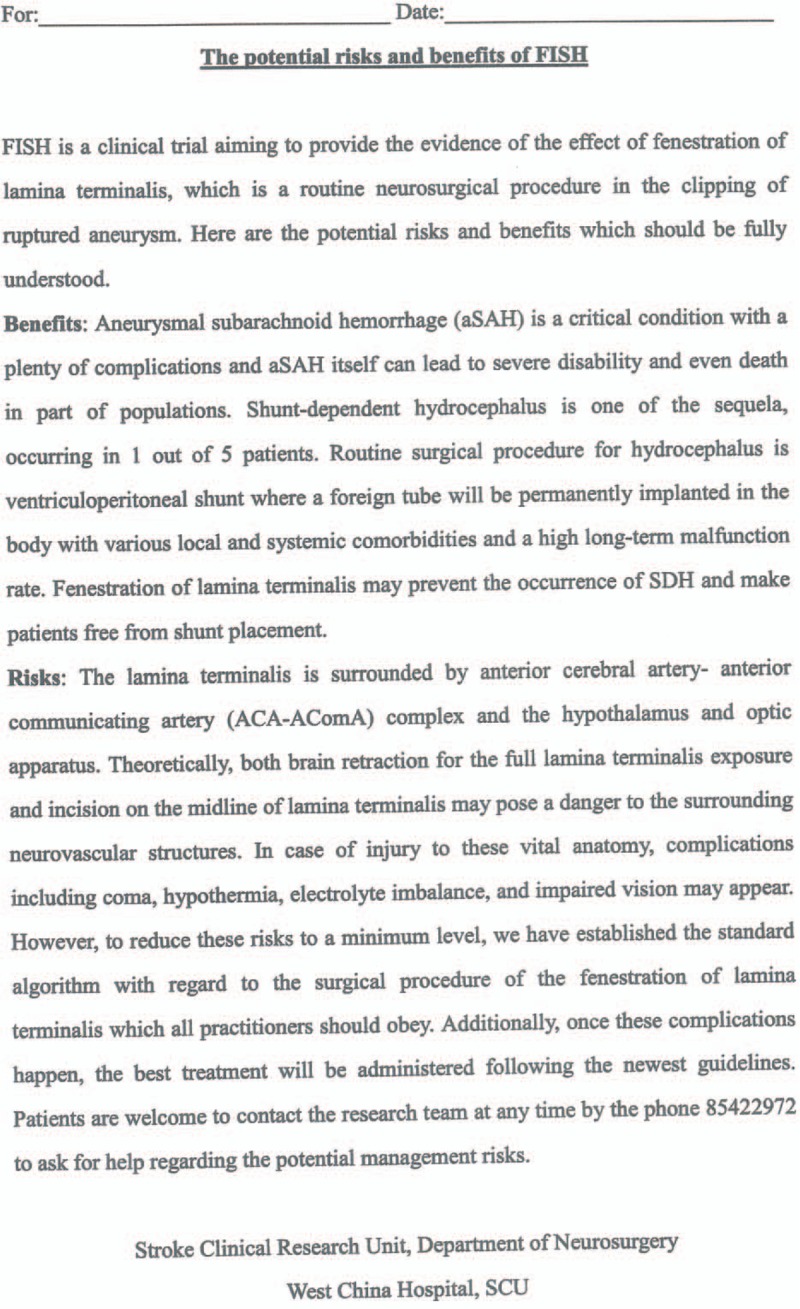
The potential risks and benefit of FISH.

This study will be conducted at the Department of Neurosurgery of West China Hospital, Sichuan University. The clinical trial has been approved by the Biological and Medical Ethics Committee (BMEC) of West China Hospital (2016-No.197) and has been registered in the Chinese Clinical Trial Registry (ChiCTR-INR-16009249). All patients or their relatives will be fully notified including the potential benefits and risks of this trial (Fig. 2) and informed consent will be achieved. Withdrawal from the trial is allowed at any point without any constraint for each subject.

### 2.3 Recruitment, randomization, and blinding

#### 2.3.1 Recruitment

West China Hospital is a 4300-bed, superior large comprehensive researching and teaching hospital in China. Our neurovascular center consists of 5 special teams and more than 600 aneurysm surgical clipping are performed annually. All potential subjects in hospital are encouraged to participate in the FISH trial. Because of the sufficient patients’ resources, adequate participant enrolment to reach target sample size is expected within 1 year at most.

#### 2.3.2 Randomization

Patients will be allocated to either single aneurysm clipping group or aneurysm clipping plus FLT group by the minimization method that is performed by a minimization software. The stratification factors for minimization include age, Fisher grade, admission GCS, and acute hydrocephalus. Once a patient is qualified, the baseline information including demographics, medical history, time from SAH onset, Hunt–Hess grade, Fisher grade, and physical examination of the patient will be sent to a special researcher who is in charge of patients randomization.

#### 2.3.3 Blinding

In this trial, because both the patients and doctors cannot be blinded, the assessor must be blinded to guarantee the research quality. Other measures are further adopted. First, the outcomes will be evaluated by 2 independent investigators who participate in neither the allocation nor the treatment and the final results will be filled in electronic case reports forms (CRF) without any modification once they are confirmed. Second, patients and related medical staff will be instructed not to leak any information regarding the treatment before and during the outcome evaluation. In addition, a statistician who will perform the statistical analysis is not allowed to take part in the implementation of the clinical trial (Fig. 2).

### 2.4 Eligibility criteria

#### 2.4.1 Inclusion criteria

1. Age ≥40 years but less than 70 years;
2. SAH confirmed by head CT scan or lumbar puncture;
3. Computed tomography angiography (CTA) and digital subtraction angiogram (DSA)-proved diagnosis of anterior circulation aneurysm;
4. Admission within 72 hours from stroke ictus;
5. SAH is graded as Fisher grade 3–4 on admission by initial CT scan;
6. Clinical symptoms categorized as Hunt–Hess grade 3–4;
7. Signed informed consent.

#### 2.4.2 Exclusion criteria

1. SAH secondary to nonaneurysmal entities such as hemorrhagic diatheses, trauma, tumor, or arteriovenous malformation, cavernous malformation;
2. Early death due to rebleeding or other complications before surgery;
3. Patients of extreme clinical severity, such as those with no spontaneous breathing, bilateral dilated pupils, or Hunt–Hess grade 5;
4. Prior hydrocephalus or ventriculoperitoneal shunt, endoscopic third ventriculostomy, or FLT;
5. Aneurysm beyond the access of surgical FLT including the distal anterior/middle cerebral aneurysms;
6. DSA-negative SAH;
7. Coagulopathy, or other known definite contraindication to operation;
8. Patients complicated with vital organ dysfunction;
9. Patient with poor compliance;
10. Pregnant;
11. Prior dementia or modified Rankin score (mRS) ≥3.

### 2.5 Intervention

Patients allocated in the control group will undergo standard microsurgical aneurysm clipping via pterion approach within 3 days after stroke onset. Sharp dissection of sylvian cistern, and then carotid, chiasmatic cisterns will be made to decrease intracranial pressure and expose the aneurysm according to the preoperative angiography. The Liliequist membrane will also be opened. The neck of aneurysm is seen clearly and clipped correctly avoiding the near perforating artery occlusion and parental artery narrowing under microscope of high resolution. The subarachnoid clot around the basal cistern will be meticulously cleared away. We do not routinely perform ventriculostomy that is reserved for patients with decreased consciousness and acute hydrocephalus.

Patients in the interventional group will undergo an additional fenestration of laminal terminalis after the same aneurysm clipping procedure. FLT will be performed in the following steps: a self-retaining retractor adjusted parallel to the anterior communicating artery to completely expose the lamina terminalis that is a bluish, bulging membrane behind the optic chiasm; an incision about 5 mm in length made strictly in the midline of the lamina terminalis to avoid injury to the adjacent neurovascular structures; the opened rim briefly coagulated to avoid subsequent reclosure as a result of adhesions. Postoperatively, standard medical treatment will be used in accordance with the recommendation of the professional guideline.

The trial will be terminated in advance if apparent benefit or higher rate of severe AE in one of the 2 interventions is revealed at a high significance level that is defined as differences of more than 3 standard deviations. To improve the adherence of protocol intervention, surgeons are not allowed to change the interventional strategy once the randomized grouping is determined. In addition, the video record of the entire surgical process will be checked at random to monitor the consistency of intervention. External CSF drainage is prohibited during the operation in the trial.

### 2.6 Outcomes

#### 2.6.1 Primary outcome

The primary outcome of this trial is the incidence of shunt-dependent hydrocephalus confirmed by both clinical symptoms and CT scan at 1 year after operation. Clinical manifestation includes a progressive decline in neurological function (deterioration of mental status, urinary incontinence, gait disturbance, and memory impairment). The radiographic diagnosis of hydrocephalus is made on the basis of the ventriculomegaly with the following observations: enlarged frontal and temporal horns, enlargement of the third ventricle, a narrowed pattern of cortical sulci, and the presence of periventricular oozing. Ventricular size is determined by the cerebral ventricular index (the greatest width between the frontal horns at the level of the Monro foramen divided by the transverse inner distance of the cranium at the same level).^[26]^ Radiologically, hydrocephalus is diagnosed when the ventricular size exceeds the 95th percentile after adjusting for patient age. Whether or not shunt is placed depends on the following criteria: ventricular enlargement on radiological imaging stated above, the progressive neurological dysfunction, and the consequent improvement after lumbar drainage.^[31]^

#### 2.6.2 Secondary outcomes

The major secondary outcomes are short-term and long-term neurological outcomes at 3 months and 1 year, respectively. The unfavorable outcomes indicate death and dependency which are defined as the modified Rankin Scale (mRS) score ≥3. Others will include the incidence of cerebral vasospasm and delayed cerebral ischemia within 1 month, the systematic complications, and days of hospitalization.

#### 2.6.3 Sample size

The rate of SDH after anterior circulation aneurysm clipping was reported to range from 9.8% to 36% with an average rate of about 20% ,^[3,9,21]^ while the incidence of SDH was less than 5% in patients undergoing fenestration during aneurysm repair.^[8]^ We hypothesize that FLT can reduce the incidence of SDH from 20% to 5%. When statistical power is chosen to be 80% and a significant level to be 0.05 with a consideration of 10% loss to follow-up, a total of 288 patients are yielded with 144 patients in each arm.

### 2.7 Statistical analysis

The “intention to treat” analysis method will be adopted in this study. A simple comparison for rates of SDH, mortality, and cerebral vasospasm will be made by *χ*^2^ test or Fisher exact test. The functional outcomes will be analyzed by Mann–Whitney *U* test. The log-rank method will be used with regard to the analysis of time-to-event type of outcomes. Preset subgroup (including age, location of aneurysm, WFNS grades, Fisher grade, time to randomization, time to operation) analysis will be performed. We will use logistic regression model to adjust the effect of multiple variables.

### 2.8 Data collection and management

The trialist who specializes in randomization will store the allocation data and neurosurgeons will fill in CRF. The follow-up information form must be separately finished by another 2 investigators who have been trained eligible for outcome evaluation. We will use double input method for the data entry and the database will be inspected by the Quality Monitoring Board (QMB) and the principal investigator, then be locked up and sent to the statistician.

### 2.9 Data safety and monitoring board (DSMB)

A DSMB of Stroke Clinical Research Unit at West China Hospital, Sichuan University, will independently monitor the safety and efficacy of this trial. The DSMB consists of an independent neurosurgeon, a neurologist, and a neuroradiologist. Members of DSMB will meet personally or by telephone once every half-year to review the relevant data including dropout rates and adverse events (AEs).

AE is determined by any unexpected incidents of the patients enrolled during the trial. Severe adverse effect (SAE) is defined as death or persistent vegetable state. All AEs and SAEs should be documented in the CRF in detail, including a description of the event, the association with the trial, the beginning and ending time, actions taken as well as their efficacy. Severe disability is excluded from SAE as aSAH can cause severe disability in a certain proportion of survivors. The DSMB should be notified within 24 hours in case of the occurrence of SAEs.

### 2.10 Study organization and funding

This study is conducted by the Department of Neurosurgery of West China Hospital, Sichuan University, supported by the National Natural Science Foundation of China (No. 81601155 and No. 81400955).

### 2.11 Protocol amendment and data dissemination

Only approved by BMEC, can any protocol amendments be done before the clinical implementation. Only relevant identifying information from participants will be collected, stored, and well protected. All researchers have been trained in the topic of confidentiality, understanding that information access should be restricted only for the purpose of scientific research. The final data from this trial will be publicly published in a peer-reviewed journal according to CONSORT guidelines.

## 3 Discussion

The FISH trial is the first large randomized, parallel-arm clinical trial, aiming to provide the robust evidence for the clinical practice by evaluating the efficacy of FLT in preventing SDH after acute aSAH. The time of our follow-up was designed on the basis of data, suggesting that more than 90% of SDH occur within 1 year after aSAH.^[9,17]^ A total of 288 patients will be eligibly enrolled in this clinical trial. The primary outcome is the incidence of SDH at 1 year. We also investigate the incidence of vasospasm within 1 month, the short-term and long-term functional outcomes as well as the mortality. In view of the simple surgical procedure with less postsurgical complications and the relatively high incidence of SDH and SDH-induced severe neurological impairment after aSAH, the FISH trial is urgently needed and the results are worth waiting.
